# Evolutionary Relationships Among Barley and *Arabidopsis* Core Circadian Clock and Clock-Associated Genes

**DOI:** 10.1007/s00239-015-9665-0

**Published:** 2015-01-22

**Authors:** Cristiane P. G. Calixto, Robbie Waugh, John W. S. Brown

**Affiliations:** 1 Division of Plant Sciences, University of Dundee at the James Hutton Institute, Invergowrie, Dundee, DD2 5DA UK; 2 Cell and Molecular Sciences, The James Hutton Institute, Invergowrie, Dundee, DD2 5DA UK

**Keywords:** *Arabidopsis thaliana*, *Hordeum vulgare* (barley), Circadian clock, Reciprocal BLAST, Homologue

## Abstract

The circadian clock regulates a multitude of plant developmental and metabolic processes. In crop species, it contributes significantly to plant performance and productivity and to the adaptation and geographical range over which crops can be grown. To understand the clock in barley and how it relates to the components in the *Arabidopsis thaliana* clock, we have performed a systematic analysis of core circadian clock and clock-associated genes in barley, Arabidopsis and another eight species including tomato, potato, a range of monocotyledonous species and the moss, *Physcomitrella patens*. We have identified orthologues and paralogues of Arabidopsis genes which are conserved in all species, monocot/dicot differences, species-specific differences and variation in gene copy number (e.g. gene duplications among the various species). We propose that the common ancestor of barley and Arabidopsis had two-thirds of the key clock components identified in Arabidopsis prior to the separation of the monocot/dicot groups. After this separation, multiple independent gene duplication events took place in both monocot and dicot ancestors.

**Electronic supplementary material:**

The online version of this article (doi:10.1007/s00239-015-9665-0) contains supplementary material, which is available to authorized users.

## Introduction

**Fig. 1:**
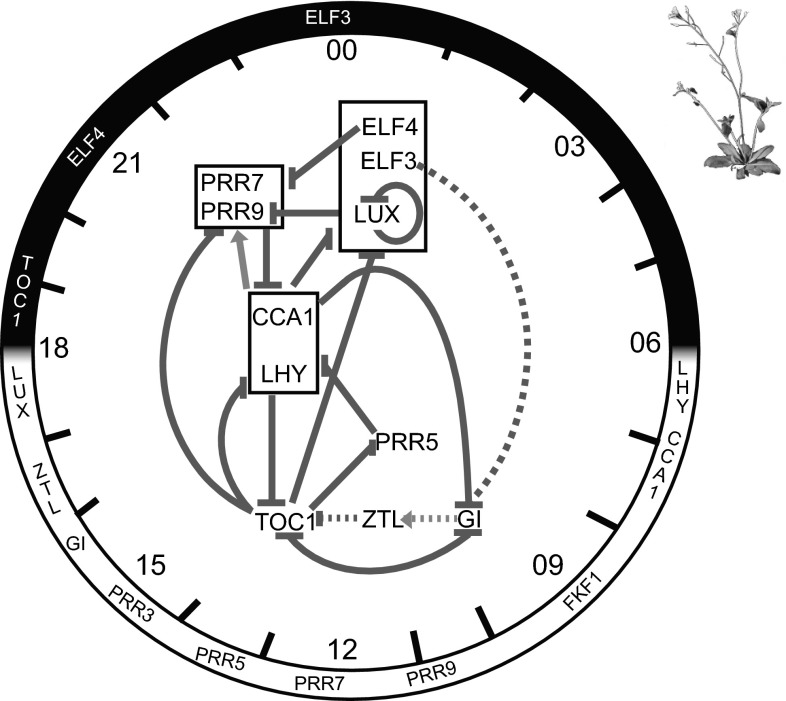
Feedback loops of the Arabidopsis clock. Simplified schematic diagram of the 24-h Arabidopsis clock. Feedback loops of the core clock genes are represented in the *centre*. *Full lines* represent transcriptional feedback loops, whereas *dashed lines* represent post-translational regulation. *Arrows* represent activation, while *arrows with blunt ends* represent repression. The diagram represents a compilation of gene regulation from numerous publications referred to in the “Introduction”. For simplicity, the PRR3 component was not included in the above regulatory network. Expression peaks of clock genes are represented at different times of the day and night in the *outer circle* (Nakamichi 2011)

Most living organisms optimise their day/night responses by measuring time and using this information to organize their physiology and morphology in anticipation of daily changes (Chen and McKnight 2007; Green et al. 2002; Okamura 2004). As sessile organisms, plants also rely on the circadian clock to optimise several physiological processes, such as expression of chlorophyll biosynthetic genes after dawn, to optimise chlorophyll content and carbon fixation (Dodd et al. 2005; Harmer et al. 2000; Haydon et al. 2013). The diversity of processes controlled by the circadian clock also reflects the number of genes under its control. Expression of about one-third of the Arabidopsis genome is regulated by the circadian clock (Covington et al. 2008). Only a relatively small number of genes establish and maintain the circadian rhythm of the clock. These core clock components are present in each cell and consist of a complex network of genes regulated by transcriptional feedback loops, post-transcriptional and post-translational modifications (Gallego and Virshup 2007; James et al. 2012; McClung 2014; Sanchez et al. 2010; Troein et al. 2009) (Fig. 1). The framework of the Arabidopsis circadian clock known as the interlocking-loop model comprises at least three interlocking gene expression feedback loops (Harmer 2010; Locke et al. 2006; Pokhilko et al. 2010; Zeilinger et al. 2006).

The central loop is formed by *CIRCADIAN CLOCK ASSOCIATED 1* (*CCA1*), *LATE ELONGATED HYPOCOTYL* (*LHY*) and *TIMING OF CHLOROPHYLL A/B BINDING PROTEIN 1* (*TOC1*—also known as *PSEUDO RESPONSE REGULATOR 1*, *PRR1*) (Alabadí et al. 2001). CCA1 and LHY are closely related and partially redundant myeloblastosis (MYB) transcription factors that accumulate at dawn and bind to the promoter region of *TOC1*, inhibiting its expression. Recent studies suggest that TOC1 is responsible for reducing *CCA1* and *LHY* expression (Gendron et al. 2012; Huang et al. 2012; Pokhilko et al. 2012). During the morning, CCA1 and LHY play parallel roles in the central loop by inducing expression of the transcriptional repressors PSEUDO RESPONSE REGULATOR 7 and 9 (PRR7 and PRR9), which along with PSEUDO RESPONSE REGULATOR 5 (PRR5) inhibit expression of *CCA1* and *LHY* (Locke et al. 2006; Nakamichi et al. 2010; Zeilinger et al. 2006). This molecular link between *CCA1*/*LHY* and *PRR7/9/5* during the morning constitutes a second feedback loop called the ‘morning loop’.

Further regulatory clock control is carried out by CCA1 and LHY through transcriptional inhibition of *EARLY FLOWERING 3* and *4* (*ELF3* and *ELF4*), *LUX ARRHYTHMO* (*LUX*, also known as *PHYTOCLOCK* *1*, *PCL1*), and *GIGANTEA* (*GI*) genes (Nagel and Kay 2012). In the ‘evening loop’, TOC1 represses expression of *PRR5*, *PRR7*, *PRR9*, *LUX*, *GI* and *ELF4* (Gendron et al. 2012; Huang et al. 2012). An important component of the evening loop is the Evening Complex (EC). The EC is composed of EARLY FLOWERING 3 (ELF3), ELF4, and LUX and it represses transcription of *PRR9* (Chow et al. 2012). Interestingly, LUX represses its own expression (Helfer et al. 2011). Further post-translational regulation takes place in the evening, such as GI degradation by ELF3 (Yu et al. 2008) and F-box protein ZEITLUPE (ZTL) stabilisation by GI, allowing ZTL to control TOC1 protein degradation (Kim et al. 2007).

The circadian clock can be entrained by certain cues, for instance light (photoperiod) and temperature (Hotta et al. 2007), which is tightly linked to plant adaptation to specific environments (Michael et al. 2003). To address the impact of the clock in crop species, such as barley, one approach is to gain an understanding of key clock components and their interactions by examining how widely clock genes are conserved. Most information on plant circadian clocks is available for Arabidopsis (Nagel and Kay 2012; Nakamichi 2011). Translation of knowledge will not be straight forward due to differences in clock control between monocots and Arabidopsis, such as rhythmicity of growth (Matos et al. 2014; Poiré et al. 2010) and different versions of the clock operating in different parts of the plant (Endo et al. 2014; James et al. 2008). Understanding the evolutionary relationships among clock genes will aid the development of clock models for other species but it is important to note that the identification of barley homologous genes does not necessarily imply conserved clock function. To date, some clock genes have been identified in monocots such as *Brachypodium distachyon* (Higgins et al. 2010) and *Zea mays* (Wang et al. 2011) with most information on rice (Hayama et al. 2003; Higgins et al. 2010; Iwamoto et al. 2009; Murakami et al. 2007; Onai and Ishiura 2005; Shin et al. 2004). For barley, circadian rhythms have been observed at diverse levels including at transcript and protein abundance, and physiological processes (Lillo 2006; Martínez et al. 2003; Nagasaka et al. 2009; Vallelian-Bindschedler et al. 1998). Diurnal and circadian expression analyses have been reported for *HvLHY* (*HvCCA1*), *HvPPD*-*H1,* *HvPRR73*, *HvPRR59*, *HvPRR95*, *HvGI*, *HvTOC1*, *HvLUX* and *HvELF3* (Campoli et al. 2012b, 2013; Dunford et al. 2005; Faure et al. 2012; Higgins et al. 2010; Turner et al. 2005). Only three barley clock genes have been well characterised using mutant plants: *Ppd*-*H1, ELF3* and *LUX* (Campoli et al. 2013; Faure et al. 2012; Turner et al. 2005; Zakhrabekova et al. 2012). The *Ppd*-*H1/PRR37* allele is the major determinant of photoperiod response in barley and is the putative *AtPRR7* orthologue (Turner et al. 2005). Mutations in the barley *Ppd*-*H1/PRR37* (*PRR7)* and *ELF3* genes affect important traits, such as flowering time (Faure et al. 2012; Stracke et al. 2009; Turner et al. 2005; Zakhrabekova et al. 2012) and low-temperature tolerance (Fowler et al. 2001).

The availability of high-confidence barley gene sequences (Matsumoto et al. 2011; Mayer et al. 2012) now allows the identification of barley orthologues of clock and clock-associated genes. Here we have performed a systematic analysis of clock genes in ten different plant species and thereby identified the genomic sequences of 21 putative barley homologues of Arabidopsis core circadian clock genes and selected clock-associated genes and propose an evolutionary history for barley and Arabidopsis clock genes from a common ancestor.

## Materials and Methods

### Cross-Species Reciprocal BLAST

To identify plant orthologues of the Arabidopsis clock genes, systematic cross-species reciprocal BLAST searches were performed using default settings and gene sequences of ten different plant species: Arabidopsis, tomato, potato, barley, *Brachypodium distachyon*, sorghum, wheat, maize, rice and moss (*Physcomitrella patens*) (Table S1). First, a BLAST search (Altschul et al. 1990) was carried out using Arabidopsis gene sequences against various databases (Table S1) to identify putative orthologous sequences. Next, reciprocal BLAST analysis was performed using the top hit from all species against the Arabidopsis database. Subsequently, cross-species reciprocal BLAST analysis was performed using the top hit from all species against each species’ databases. When the top hit of a reciprocal BLAST successfully identified the original Arabidopsis sequence and the top hits from all other databases, these were taken as orthologues. Any additional hits with an *E*-value similar to the top hit were also subjected to reciprocal BLASTs. When the second/third/etc. best hits successfully identified the original Arabidopsis sequence and their orthologues in all other species, these were taken as paralogues.

However, when a reciprocal BLAST with the top hit identified a different Arabidopsis gene from the original candidate sequence, (1) the newly identified Arabidopsis gene(s) was used in cross-species reciprocal BLAST analysis; and (2) all gene family members of the new and original Arabidopsis candidate genes were also subjected to cross-species reciprocal BLASTs. Similarly, in this analysis with ‘additional’ Arabidopsis sequences, when the top hit of a cross-species BLAST reciprocally identified the top hit from another species, these were taken as orthologues. This analysis identified genes in Arabidopsis which were related to the initial candidate clock gene and their putative orthologues in other species. These cross-species reciprocal BLAST analyses of ‘additional’ Arabidopsis genes also considered any additional hits with *E*-value similar to the top hit, subjecting them to cross-species reciprocal BLASTs (as mentioned above). Overall, these analyses identified true orthologues and duplicated genes in the tested species.

Gene sequences and identifiers were taken from the databases described in Table S1. Schematic diagrams of genomic structures were initially made using the Exon–Intron Graphic Maker program (http://wormweb.org/exonintron). In some cases, the annotated exon/intron gene structures did not generate full length ORFs, when compared to homologous genes. Therefore, when necessary, re-annotation of genomic sequences was performed based on: (1) cDNA, EST and PUT (PlantGDB-assembled Unique Transcripts) data available for the related species; (2) the presence of GT and AG dinucleotides for intron boundaries (5′ and 3′ splice site, respectively); (3) ORF maintenance of each exon; and (4) the annotation of orthologous mRNA/protein sequences.

### Phylogenetic Analysis

Nucleotide sequence alignments were performed such that they preserved the codon structure of putative coding sequences (CDS). For this, nucleotide alignments were based on the alignments of their deduced protein sequence using the ClustalW program (Larkin et al. 2007; Tamura et al. 2013). Gene tree estimation was performed using the neighbour-joining (NJ) method (Saitou and Nei 1987) available on MEGA6 software (Tamura et al. 2013). The moss *P. patens* was used as an outgroup for angiosperm species, and moss genes, when present, were used to root the phylogenetic trees. Statistical support for each branch on phylogenetic trees was generated from the bootstrap test (2,000 replicates; values shown when >50 %) (Felsenstein 1985). The evolutionary distances and branch lengths were computed using the Maximum Composite Likelihood method (Tamura et al. 2004). Pseudogenes were not analysed in order to prevent poorly supported topologies on reconstruction of phylogeny from gene families, as suggested by Zimmer et al. (2007).

## Results

### Identification of Barley Core Clock and Clock-Associated Genes by Reciprocal BLAST

The Arabidopsis clock and clock-associated genes, including selected flowering-related genes: *CCA1*, *LHY*, *TOC1 (PRR1)*, *GI*, *ELF3*, *ELF4*, *PRR7*, *PRR3*, *PRR9*, *PRR5*, *LUX (PCL1)*, *FKF1*, *ZTL*, *CHE (TCP21)*, *GRP7 (CCR2)*, *GRP8*, *CAB2*, *CO* and *FT* were selected for a comparative approach to identify and confirm the genomic sequences of related genes in barley. Barley and Arabidopsis share a common ancestor but they have diverged considerably since their separation around 140 million years ago (Mya) (Chaw et al. 2004; Moore et al. 2007). Since orthology determination becomes more difficult when species are evolutionarily distant (Prosdocimi et al. 2009; Yu and Hinchcliffe 2011), additional species with whole genome sequence information from both dicot and monocot groups were included in the comparative analysis. These species were tomato, potato, moss (*P. patens*) and another five grasses: *Brachypodium distachyon*, sorghum, wheat, maize and rice (Table S1). The comparative approach comprised multiple cross-species reciprocal BLASTs (Altschul et al. 1990) as described in “Materials and methods”. These systematic analyses identified the range of species which contained true orthologues and a comprehensive list of the duplicated genes in the analysed species (Table 1, S2–S6). In a few cases, false duplicated genes, previously described in the literature, are described in Supplementary Note 1.

**Table 1:** Circadian clock and clock-associated genes in* Arabidopsis* and their barley homologues

*Arabidopsis* homologues			Barley homologues		
Paralogues	Orthologues/Paralogues^a^	Orthologues	Orthologues	Orthologues/Paralogues^a^	Paralogues
AtCCA1 (At2g46830)	–	AtLHY (At1g01060)	HvLHY (MLOC_14118)	–	–
AtBOA (At5g59570)	–	AtLUX (At3g46640)	HvLUX (MLOC_37446)	–	–
EEC?	–	AtELF3 (At2g25930)	HvELF3 (MLOC_78552^b^)	–	–
–	–	AtGI (At1g22770)	HvGI (MLOC_70638^b^)	–	–
–	–	AtTOC1 (At5g61380)	HvTOC1 (MLOC_52387)	–	–
–	AtPRR5 (At5g24470) AtPRR9 (At2g46790)	–	–	HvPRR95 (MLOC_57021) HvPRR59 (MLOC_62596^b^)	–
AtPRR3 (At5g60100)	–	AtPRR7 (At5g02810)	HvPpd-H1 (MLOC_81154)	–	HvPRR73 (MLOC_12732)
AtLPK2 (At2g18915)	–	AtZTL (At5g57360)	–	HvZTLa (MLOC_44010) HvZTLb (MLOC_20007)	–
–	–	AtFKF1 (At1g68050)	HvFKF1 (MLOC_53725)	–	–
AtGRP8 (At4g39260)	–	AtGRP7 (At2g21660)	–	HvGRP7a (MLOC_17819^b^) HvGRP7b (MLOC_59695^b^)	–
At3g02380 (AtCOL2)	At5g15840 (CO) At5g15850 (COL1)	–	–	HvCO1 (MLOC_6921^b^) HvCO2 (MLOC_75496^b^)	–
AtTSF (At4g20370)	–	AtFT (At1g65480)	–	HvFT1 (MLOC_68576) HvFT2 (MLOC_10172^b^)	–
–	At2g40080 (ELF4) At2g29950 (ELF4-like1)		–	–	–
At1g17455 (ELF4-like4) At1g72630 (ELF4-like2)	–	At2g06255 (ELF4-like3)	HvELF4-like3 (MLOC_70937)	–	HvELF4-likeA (MLOC_58590)

^a^Determination of one-to-one gene orthologue/paralogue not defined

^b^MLOC represents partial sequence of the gene

**Fig. 2:**
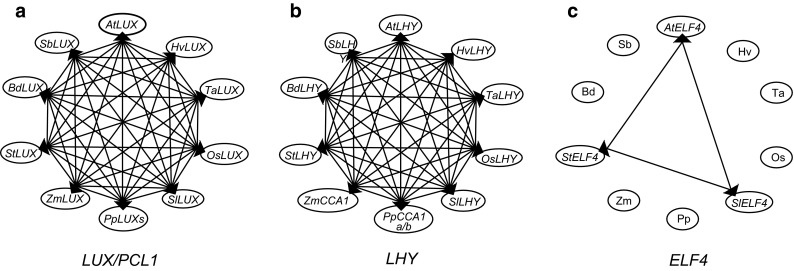
Robust analysis in the identification of clock orthologues. Cross-species reciprocal BLAST diagram of **a** *LUX*, **b** *LHY* and **c** *ELF4* genes. *Arrows* indicate direction of BLAST analysis, i.e. a sequence from one database was used to identify orthologous sequences in the database of another species

The Arabidopsis clock genes showed variation in their ability to identify true orthologues providing some information on the clock gene components in different species and their evolution. This is illustrated by considering genes with very different results from the analysis: *LUX*, *LHY*/*CCA1* and *ELF4*. *AtLUX* identified true orthologues in all nine species analysed by cross-species reciprocal BLAST, including another paralogue in Arabidopsis (*AtBOA*) and four gene copies in *P. patens* (Fig. 2a; Table S2). The latter species also has a number of particular features regarding its clock flowering-related genes where *GI*, *FKFI*, *ZTL*, *CO* and *FT* are present in all flowering plants but absent in *P. patens* (Tables S2, S4 and S6). At the other extreme is *AtCCA1*. This gene identified a gene in each of the nine species but it had no reciprocal hits with any species analysed. In fact, the reciprocal BLASTs all identified *AtLHY* instead of *AtCCA1*. When *AtLHY* was used, cross-species reciprocal BLASTs were successful with all ten species (Fig. 2b) suggesting that they contained true orthologues of *AtLHY* but no orthologues of *AtCCA1*. Therefore, barley and six other plants have a single *LHY* counterpart, whereas *LHY* gene duplications possibly occurred independently in maize, *P. patens* and Arabidopsis, the latter giving rise to *AtCCA1*.

Other genes, for example *ELF4*, only had cross-species reciprocal hits with dicot species suggesting that it is specific to dicots (Fig. 2c). In this analysis, the initial BLAST using the *AtELF4* sequence identified sequences in monocots that did not identify *AtELF4* reciprocally but instead identified *AtELF4*-*like3*. Using this gene and all known *AtELF4* gene family members, orthologues and paralogues of *ELF4*-*like3* genes in all species analysed were identified (Tables S5 and S6). Barley and wheat each have two genes in this family. Cross-species reciprocal BLAST using the single-exon genes *AtCHE* and *AtCAB2* did not identify orthologues in any of the species analysed (Supplementary Note 2).

### Genomic Structure of Barley and Arabidopsis Clock Genes

Having identified barley orthologues of clock genes, we were then able to examine the conservation of exon–intron organisation to gain further support for the relationships between orthologues. Genomic sequences of genes related to Arabidopsis clock genes were downloaded from the various plant databases for analysis and correctly annotated or re-annotated as necessary. The 21 genes which were (re)annotated are shown in Tables S2–S6.

**Fig. 3:**
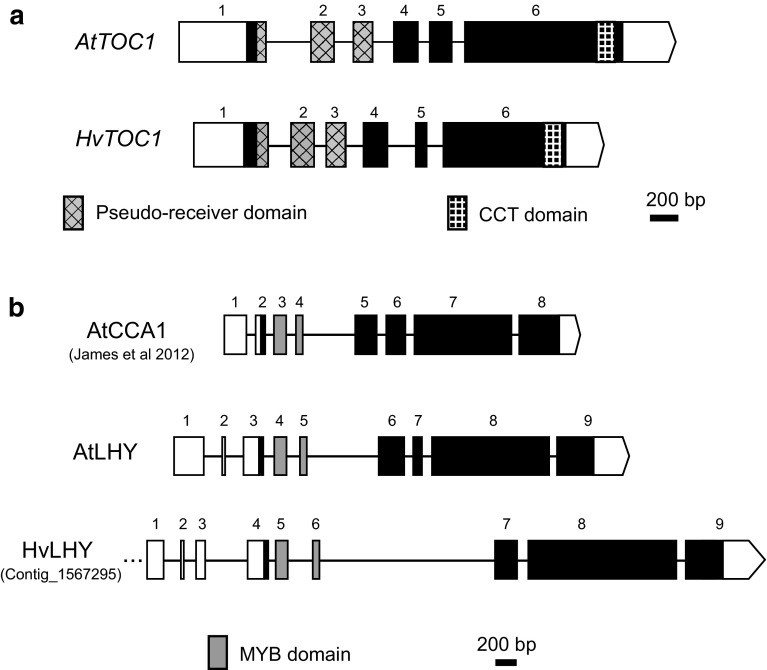
Genomic structure of **a** TOC1 (PRR1) and **b** *LHY* and *CCA1* in *Arabidopsis* (At) and barley (Hv). Exons are numbered; 5′ and 3′ UTRs are *open boxes*; coding sequences are *dark boxes*, except domain-encoding exons. There may be further 5′ UTR sequence upstream of the *HvLHY* exon 1 designated in the Figure (*dotted line*) which has not yet been fully sequenced

The genomic structures of barley and Arabidopsis genes are generally well conserved in their exon/intron organisation (e.g. *TOC1* in Fig. 3a). However, differences in the barley orthologues are mainly in the size of introns, which are generally much larger in barley, and in the UTR sequences. A clear example is the 5′ UTR of *LHY* in barley, which is considerably longer and has a complex multi-exon structure, while *AtLHY* only has two 5′ UTR introns (Fig. 3b). In the coding region, *AtCCA1*, *AtLHY* and *HvLHY* have a highly conserved gene structure, with the exception of one additional intron found in *AtCCA1* and *AtLHY* (intron 5 or 6, respectively) when compared with *HvLHY* (Fig. 3b). The genomic structures of *HvPRR37*/*Ppd*-*H1*, *GI* and *ELF3* have been analysed previously (Dunford et al. 2005; Turner et al. 2005; Zakhrabekova et al. 2012). An important consideration remains that the barley gene space is not complete (Mayer et al. 2012) and the extensive in silico analysis conducted here may still have missed possible orthologues or parts of genes (e.g. the 5′ UTRs of *HvLHY* and *HvPRR95)*.

### Phylogenetic Analyses of Clock Genes

**Fig. 4:**
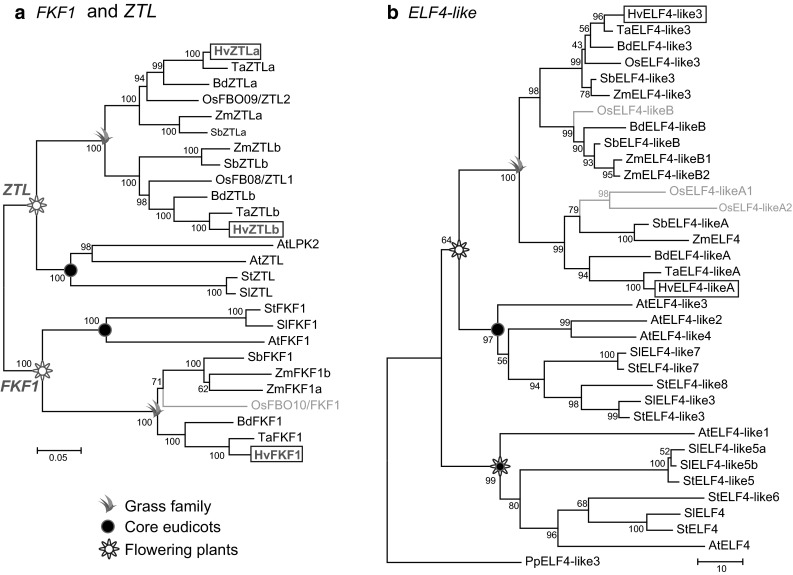
**a** Phylogenetic tree of *ZTL* and *FKF1* genes. Due to the lack of complete sequence information for the *TaZTLb* gene, the partial wheat *ZTLb* CDS from PUT43520 was used to represent wheat. Since *P. patens* does not contain a true orthologue of *ZTL* or *FKF1*, the root was placed on the *FKF1* family branch. **b** Phylogenetic trees of the *ELF4*-*like* family. Due to the lack of complete CDS data for the *TaELF4*-*like3*, the partially related cDNA from PUT145474 was used to represent this wheat branch. In constructing the trees, all gaps and missing data were eliminated from sequence alignments. Genes that do not follow expected topology are shown in *grey*. Evolutionary distances are presented in number of base substitutions per site. Barley genes are *highlighted with a box*

To demonstrate and confirm the degree of relatedness of identified orthologous genes, phylogenetic trees were generated (Fig. 4a, b; Figs. S1–S3).

#### *ZTL* and *FKF1* Orthologues

Gene members of the LOV (light, oxygen or voltage) blue light receptor subfamily, *ZTL* and *FKF1*, were identified in all flowering plants analysed (Fig. 4a). In Arabidopsis, *FKF1* is functionally and evolutionary diverged from *ZTL*, which might have started sometime after euphyllophyte (ferns and seed plants) speciation (Suetsugu and Wada 2013). The *ZTL* gene has been duplicated in both the ancestor of monocots and in Arabidopsis. As a result, monocots have *ZTLa* and *ZTLb* genes, while Arabidopsis has *ZTL* and the recent copy, *LPK2* (Lou et al. 2012). The exact relationships between both monocot *ZTL* genes and the dicot *ZTL* could not be determined, i.e. the true orthologue of *AtZTL* in monocots is either *ZTLa* or *ZTLb*. Monocots and dicots have maintained a single copy of *FKF1* except for maize, which has two copies due to a recent duplication.

#### *ELF4* and *ELF4*-*like3* Orthologues

The in silico analyses suggest two subgroups for the *ELF4*-*like* family: *ELF4*, which includes *AtELF4*-*like1* (Table S5), and *ELF4*-*like2/3/4* (Table S6). *ELF4* family members are found only in dicot species and they are single-exon genes. *ELF4*-*like2/3/4* family members are found in all plants analysed and most of them have a 5′ UTR intron. Our analyses suggest that the ancestor of land plants contained one copy of the *ELF4*-*like* gene, most likely an orthologue of *AtELF4*-*like3*. This gene was duplicated in the ancestor of flowering plants, which then contained both *ELF4*-*like3* and the new copy, *ELF4*. Monocots lost the *ELF4* gene, while dicot species duplicated this gene multiple times (Fig. 4b). The *ELF4*-*like3* gene was duplicated twice in monocots, but barley and wheat may have lost one of the copies. Dicots also had one or two duplication events from the *ELF4*-*like3* gene and its subsequent copies.

#### *PRR* Orthologues

Most flowering plants analysed have five *PRR* genes. The *TOC1* gene is duplicated in maize and both *Solanum* species. *P. patens* has four *PRR*s, which are very closely related to the *PRR*s of angiosperms. It was not possible to determine *PRR* orthologues due to very complex results from BLAST and phylogenetic analysis (Fig. S1). The only evidence observed is that the ancestor of flowering plants had *TOC1*, *PRR3/7* and *PRR9/5* genes. After the divergence of monocots and dicots, both ancestors independently duplicated *PRR3/7* and *PRR9/5* genes.

#### *LHY*, *LUX* and *GRP7* Orthologues

Phylogenetic analyses confirmed true orthologues of *AtLHY* (Fig. S2a), *AtLUX* (Fig. S2b) and *AtGRP7* (Fig. S2c) in all species analysed. In particular, several paralogues of the single-intron *AtGRP7* gene were identified in all species analysed. *In silico* analyses suggest that the ancestor of land plants contained one copy of the *GRP7* gene. Two independent duplication events occurred within the *P. patens* branch, generating *PpGRP1*, *PpGRP2* and Pp1s136_70. The *GRP7* gene has undergone a series of independent duplications within dicots and once in monocots. In Arabidopsis, it is likely that this duplication gave rise to *AtGRP8*, according to cross-species BLASTs. In monocots, there are two copies of the *GRP7* gene, which are hereafter called *GRP7a* and *GRP7b*. Rice has lost *GRP7a* and duplicated *GRP7b*. Wheat seems to be the only species with a third copy, *TaGRP7c*, but the predicted protein is around half the size of the other *GRP*s in monocots and may therefore be a pseudogene or an error from sequencing and consensus sequence formation, and was eliminated from further analyses.

#### *CO* and *FT* Orthologues

Homologous members of the *AtCO* subfamily were identified in all flowering plants analysed, including barley (Fig. S3a). Protein alignment and BLAST analyses suggest that the ancestor of flowering plants contained one copy of a *CO*-related gene, which is the orthologue of *AtCO* or *AtCOL1*. Two independent duplication events have occurred within the Arabidopsis branch, which currently has *AtCOL1*, *AtCO* and *AtCOL2*. Monocots have one duplication event of the original *CO*-related gene, giving rise to both *CO1* and *CO2*. Rice and maize have lost their *CO2* gene copy. The exact relationship between both *CO1* and *CO2* genes in monocots and the dicot *CO*-related genes could not be determined, but homologues are clearly present. Similarly, the true orthologue of *AtFT* in monocots could not be determined, but at least two homologues (*FT1* and *FT2*) are present in all monocots analysed (Fig. S3b). Rice in particular has two copies of the *FT1* gene (*OsFTL2* and *OsFTL3*).

#### *ELF3* and *GI* Orthologues

Homologues of *AtELF3* were identified in all species analysed. Paralogues were also observed and are probably due to a series of duplication events of the *ELF3* gene. All in silico analyses suggest that the ancestor of land plants contained one copy of the *ELF3* gene. Two independent duplication events occurred within the *P. patens* branch, which has three homologues of *ELF3*. The original *ELF3* gene was also duplicated in the ancestor of flowering plants, which then contained both the *ELF3* gene and the new copy, *ESSENCE OF ELF3 CONSENSUS* (*EEC*) gene. However, this hypothesis for the origin of *EEC* has low support from phylogenetic analysis (59 % likelihood, Fig. S3c) and must be treated with care. Monocots have lost the *EEC* gene and duplicated *ELF3*, creating the *ELF3a* and *ELF3b* genes. Temperate grasses (Pooideae) lost the *ELF3b* gene, whereas rice lost *ELF3a*. Interestingly, the *ELF3b* copy present in the rice genome has undergone a recent duplication. The exact relationships between both *ELF3* alleles in monocots and the dicot *ELF3* could not be determined. Lastly, true orthologues of *GI* were identified and confirmed in all flowering plants analysed (Fig. S3d).

In summary, we have identified the genomic sequences of 21 putative barley homologues of Arabidopsis core circadian clock genes and selected associated genes and eliminated any similar unrelated sequences, i.e. sequences that are not descended from a common ancestral sequence. A single Arabidopsis true orthologue of the clock genes *LHY*, *TOC1*, *GI*, *ELF3*, *LUX* and *FKF1* was identified in barley. Additionally, the ancestor of flowering plants possibly had a single copy of *PRR3/7*, *PRR9/5*, *FT*, *CO/COL1*, *ZTL* and *GRP7* genes and after divergence of monocots and dicots both ancestors independently duplicated and maintained these genes. Orthologues of the *AtCHE*, *AtELF4* and *AtCAB2* gene families were not identified in barley or other monocot species.

## Discussion

### *In Silico* Identification of Clock Homologues

Putative homologues of Arabidopsis circadian clock genes were identified in tomato, potato, *P. patens*, *Brachypodium*, sorghum, wheat, maize, rice and barley (Tables S2–S6). Forty of those genes in monocots, including six in barley (*HvZTLa*, *HvZTLb*, *HvGRP7b*, *HvELF4*-*like3*, *HvFKF1* and *HvCABa*), were hitherto unknown. Many genes were already known and had previously been used in simple analyses or, less commonly, a fully characterised study (see Tables S2–S6). The identification of previously described genes in various species confirmed that the in silico method used here is appropriate for identifying homologues, as well as confirming the identity of the previously described genes. Moreover, the comprehensive list of species with duplicated gene copies gives further confidence to the gene duplications identified in barley and has helped to identify some incorrect duplication events (Supplementary Note 1).

The identification of orthologous, paralogous and lost genes may provide information on the function of these genes and how they impact the growth habit of particular species. For example, *CO* and *FT* are key genes in the regulation of flowering time. *AtCO* is a member of a subfamily from Group Ia of the COL family (Griffiths et al. 2003; Valverde 2011). *In silico* analyses suggest Arabidopsis has three members from this subfamily, whereas barley has two: *HvCO1* (Campoli et al. 2012a; Griffiths et al. 2003) and *HvCO2* genes (Griffiths et al. 2003). Other monocots also have two gene copies, except rice [also suggested by Cockram et al. (2012)] and maize. These species require short day photoperiods to flower, while barley, wheat, Arabidopsis and potato, require long days. Therefore, the absence of the *CO2* gene copy in rice and maize may have had a critical role in their domestication (Cockram et al. 2012; Miller et al. 2008). Similarly, the central component in mediating the onset of flowering, the *FT* gene, was present in the angiosperm ancestor and contributed to the evolution of flowering plants (Klintenäs et al. 2012; Pin and Nilsson 2012). *AtFT* is a member of the PHOSPHATIDYLETHANOLAMINE-BINDING PROTEIN (PEBP) *FT*-like family and it forms a subfamily with *TWIN SISTER OF FT* (*TSF*) (Faure et al. 2007; Kobayashi et al. 1999). Monocots have two members from this subfamily: *FT1* and *FT2* through duplication, but neither is an orthologue of *AtTSF*. The monocot *FT1*/*FT2* duplication occurred after the divergence between the grasses and Arabidopsis. Therefore, this duplication is independent of the *FT*/*TSF* duplication in Arabidopsis, as suggested previously (Li and Dubcovsky 2008). Interestingly, *FT* copy number variation in cereals plays an important role in the regulation of plant flowering and development (Nitcher et al. 2013).

### Dicot-Specific Clock Genes

Orthologues of four Arabidopsis genes from the initial candidate list were not identified in barley and most other plant species: *ELF4*, *CAB2*, *CHE* and *CCA1*. These are likely to be dicot- or Arabidopsis-specific genes. For *ELF4*, in particular, only members of the *ELF4*-*like2/3/4* sub-clade have been found in monocots (Boxall et al. 2005; Higgins et al. 2010; Murakami et al. 2007). However, Kolmos et al. (2009) suggested that *AtELF4* and *AtELF4*-*like1* are the closest homologues of *ELF4*-*like* genes in monocots and that *HvELF4*-*likeA* fully complemented the *elf4* loss-of-function phenotype in Arabidopsis, suggesting conserved functionality (Kolmos et al. 2009). It is noteworthy that some *ELF4* family members were missing from most monocot species they analysed, which might have influenced the topology that suggested such homology. The lack of orthologues of the clock-associated genes *AtCAB2* and *AtCHE* is discussed in Supplementary Note 2.

*CCA1*, along with *LHY*, plays an important role in the regulation of the circadian rhythm in Arabidopsis, but the presence of both counterparts in the genome of other plant species does not seem to be a common feature. Barley and six other plants analysed here have only one *LHY*/*CCA1* gene, and this suggestion is also confirmed in studies of barley (Campoli et al. 2012b), rice (Murakami et al. 2007) and *Brachypodium* (Higgins et al. 2010). This raises the question of whether most species contain an orthologue of *LHY* or *CCA1*? Some analyses indicate that *LHY*, as opposed to *CCA1*, is present in most plant species (Lou et al. 2012; Takata et al. 2009; Yon et al. 2012). For instance: (1) cross-species reciprocal BLAST is possible only for *AtLHY*, not *AtCCA1*; (2) *Solanum* species have only one gene, which is very similar in sequence to *LHY*; (3) *CHE*, the transcriptional repressor of *CCA1* is also an Arabidopsis-specific gene; (4) *CCA1* is a casein kinase II (CK2) target in Arabidopsis, whereas in rice the OsCK2 orthologue does not target *OsCCA1*, probably because *OsCCA1* does not contain the correct amino acid for interaction, suggesting again that *OsCCA1* is not a true orthologue of *AtCCA1* but of *AtLHY* (Ogiso et al. 2010) and (5) *AtLHY* and *HvLHY* have similar transcriptional and post-transcriptional responses to lower temperature transitions, as opposed to *AtCCA1* behaviour (Calixto et al., manuscript in preparation). Therefore, most plant species do not contain *CCA1* and *LHY* but have only one gene, most probably *LHY*, which is necessary for maintenance of the circadian rhythm and plant survival. In Arabidopsis, where *LHY* has been duplicated, the gene copies have diverged such that both are important for the maintenance of the circadian rhythm.

### Evolution of Clock Genes

Within angiosperms, in both monocots and dicots, a strong similarity exists among their clock components, architecture and functions (Song et al. 2010). To test for evolutionary homology of monocot and dicot clock genes, several investigations have used different approaches, such as phylogenetic analysis, studies of segmental duplication and functional gene assessments through gene expression studies and complementation tests (suggesting conserved biochemical function). For example, knockdown and overexpression of *LHY*, *ELF3* and *GI* genes from *Lemna gibba* plants indicated these genes are functionally conserved with Arabidopsis and rice genes (Serikawa et al. 2008).

**Fig. 5:**
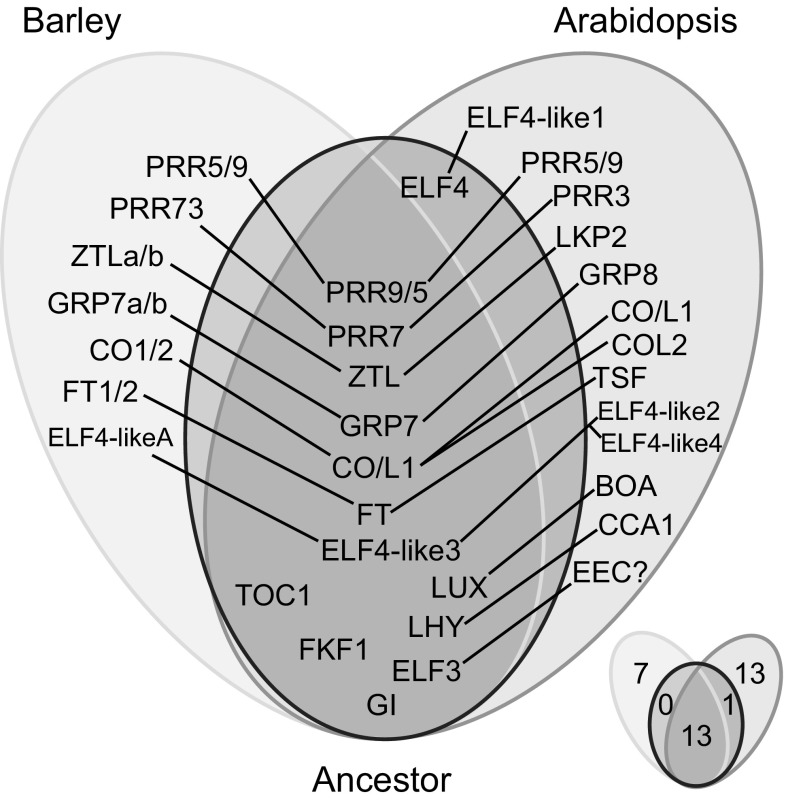
Schematic diagram of the proposed evolutionary history of circadian clock components of barley, Arabidopsis and their putative common ancestor. Independent duplication events are represented by fine *diagonal lines*. The diagram at the *bottom right* is related to the main diagram and it refers to the numbers of genes from each group

Here we propose a common evolutionary genetic history that gave rise to both barley and Arabidopsis clock genes from a common ancestor (Fig. 5). This hypothesis is based on robust in silico searches and phylogenetic analysis. Homologues of the core clock components *LHY*, *TOC1*, *PRR7* (*PRR37* in monocots), *PRR9/5*, *GI*, *LUX*, *ELF3*, *FKF1* and *ZTL* and the clock-related genes *ELF4*-*like3*, *COL1*/*CO*, *FT* and *GRP7* were present in the common ancestor of monocots and dicots. Therefore, about 60 % of barley clock genes are true orthologues of the Arabidopsis clock genes. *TOC1*, *FKF1*, *LUX* and *GI* are single copy genes for most monocots and dicots. One exception is in Arabidopsis, which has a duplicated copy of *AtLUX*, *AtBOA*. Of the core Arabidopsis clock genes, *CCA1*, *CHE* and *ELF4* are absent in barley. *ELF4*, in particular, was present in the ancestor but has been lost in monocots. As our analysis has utilised Arabidopsis clock genes as a start point, we would be unable to detect clock components present only in monocots.

Our studies with the basal land plant *P. patens* and angiosperm species suggest the circadian clock in the ancestor of land plants had a smaller set of clock genes when compared to Arabidopsis. It included the genes *LHY/CCA1*, *PRR*-*like*, *ELF3*, *LUX, GRP7* and *ELF4*-*like3* but lacked homologues of clock- and flowering-related genes *AtGI*, *AtZTL*, *AtFKF1*, *AtELF4*s, *AtCO*s and *AtFT*s which were found in all plants studied here except moss. Interestingly, the lack of those clock genes might reduce the moss clock into one single loop, from the three integrated feedback loop model of the Arabidopsis clock (Holm et al. 2010). Regarding the *PRR*-*like* gene, it is suggested that the ancestor of land plants possessed orthologues of *AtTOC1*, *AtPRR7/3*, *AtPRR9/5* and *PpPRR1/2/3/4* in its genome, but only the *PpPRR1/2/3/4* gene was maintained in the moss lineage, whereas angiosperms lost only the *PpPRR1/2/3/4* orthologue (Satbhai et al. 2010).

Multiple independent clock gene duplications have occurred in both monocot and dicot ancestors, generating paralogues. Paralogues could be functionally equivalent to missing genes (e.g. *ELF4*-*likeA*) or deviate in terms of function/regulation. *ELF3*, *ELF4*-*like3*, *FT*, *CO*/*COL1*, *GRP7*, *ZTL*, *PRR7* and *PRR9/5* were independently duplicated and maintained in both monocots and dicots, which is an interesting example of convergent evolution. In the ancestor of moss, *LHY/CCA1*, *ELF3*, *LUX*, *PpPRR1/2/3/4* and *GRP7* were independently duplicated several times as supported by studies in diverse plant species, including barley (Campoli et al. 2012b; Cockram et al. 2012; Higgins et al. 2010; Holm et al. 2010; McClung 2010; Satbhai et al. 2010). Convergent evolution also interfered with our phylogenetic analysis and the determination of one-to-one gene homology. For example, it is not certain which monocot gene, *PRR95* or *PRR59*, is the orthologue of *AtPRR9/5* (Takata et al. 2010).

A large proportion of gene duplication events has been generated by whole genome duplication (WGD) events (Paterson et al. 2010). The evolution of angiosperm genomes has been characterised by WGD events, typically accompanied by considerable gene loss (Paterson et al. 2010). However, plants have preferentially retained clock genes, which is consistent with the gene dosage hypothesis (Lou et al. 2012). This hypothesis predicts that genes encoding proteins engaged in dose-sensitive interactions, such as transcriptional or signalling networks, cannot be reduced back to single copies once all interacting partners are simultaneously duplicated in a WGD because the imbalance associated with this loss is likely to decrease fitness (Schnable et al. 2012). Additionally, paralogues could also deviate in terms of function or regulation. An example of sub-functionalisation is the *PRR3* gene in Arabidopsis, which is expressed in the vasculature (Para et al. 2007), while other *PRR*s exhibit widespread expression. An excellent example of WGD coupled with retention of dose-sensitive duplicated clock genes has recently been reported for the evolution of *Brassica* *rapa* (Lou et al. 2012). In this work, it is suggested that such phenomena have permitted the evolution of increasingly complex circadian clock mechanisms (Lou et al. 2012). Clock complexity probably allowed for increased entrainment efficiency and temporal regulation of output pathways (Tauber et al. 2004), which has contributed to adaptation of plants to different environments. In summary, the availability of the barley gene space has allowed us to identify barley clock genes and propose their evolution in relation to the model plant Arabidopsis.

## Electronic supplementary material

Below is the link to the electronic supplementary material.

Supplementary material 1 (DOCX 414 kb)
