# Supplementary material for: Evolutionary Relationships Among Barley and Arabidopsis Core Circadian Clock and Clock-Associated Genes
Source: J Mol Evol. 2015 Jan 22;80(2):108–19. doi: 10.1007/s00239-015-9665-0 (PMC4320304; doi:10.1007/s00239-015-9665-0)
Supplement: Supplementary file 1 — Supplementary material 1 (DOCX 414 kb) [file 239_2015_9665_MOESM1_ESM.docx]

*Journal of Molecular Evolution*

**Evolutionary relationships among barley and *Arabidopsis* core circadian clock
and clock-associated genes**

Cristiane P. G. Calixto^1^, Robbie Waugh^1,2^, and John W. S. Brown^1,2^

^1^Division of Plant Sciences, University of Dundee at the James Hutton Institute, Invergowrie, Dundee DD2 5DA, United Kingdom.

^2^Cell and Molecular Sciences, The James Hutton Institute, Invergowrie, Dundee DD2 5DA, United Kingdom.

Corresponding author: Prof. J.W.S. Brown

Division of Plant Sciences,

University of Dundee at The James Hutton Institute,

Invergowrie DD2 5DA, United Kingdom

Tel.: ++44-1382-568777

e-mail: [j.w.s.brown@dundee.ac.uk](mailto:j.w.s.brown@dundee.ac.uk); [John.Brown@hutton.ac.uk](mailto:John.Brown@hutton.ac.uk)

**Electronic Supplementary Material**

This Online Resource file contains Supplementary Figures S1-S3, Supplementary Tables S1-S6, Supplementary Notes 1-2 and Supplementary References.

Fig. S1 Phylogenetic tree of *PRR* genes identified by cross-species reciprocal BLAST.

**Fig. S2** Phylogenetic trees of *LHY*, *CCA1*, *LUX*, *GRP7* and *GRP8* genes.

**Fig. S3** Phylogenetic trees of *CO*, *FT*, *ELF3* and *GI* genes.

**Table S1.** The database resources of 10 plant genome sequences analysed in this work

**Table S2.** Homologues of *LHY* and *CCA1*, *LUX*, *ELF3*, and *GI* in different land plant species

**Table S3.** Homologues of the pseudo-response regulator genes *TOC1*, *PRR5(9)* and *PRR9(5)*, *PRR7(3)* and *PRR3(7)* in different land plant species

**Table S4.**  Homologues of *ZTL*, *FKF1*, *GRP7* and *GRP8* genes in different land plant species

**Table S5.**  Dicot-specific homologues of *ELF4* and *EEC* in three dicotyledonous plant species and the Arabidopsis-specific *CHE* and *TSF*

**Table S6.** Homologues of *FT*, *ELF4-like* and *CO* genes in different land plant species

**Note 1.** Likely incorrect gene duplication events

**Note 2.** Arabidopsis-specific clock-associated genes *AtCHE* and *AtCAB2*


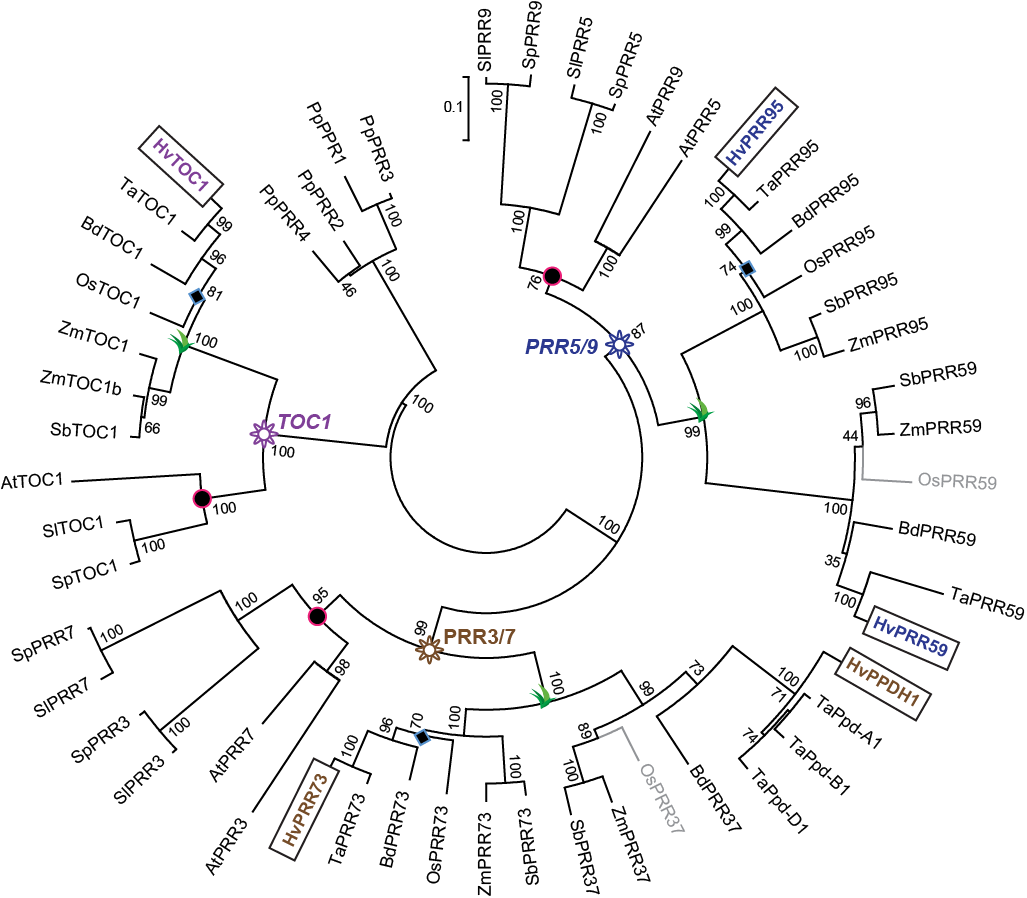


Fig. S1 Phylogenetic tree of *PRR* genes identified by cross-species reciprocal BLAST. For simplicity, *TOC1-like* sequences from tomato and potato were not included in the analysis. Alignment is based on putative mRNA sequences. Due to the lack of complete CDS data for the *TaPRR73* and *TaPRR59* genes, the partial related cDNAs from PUT18538 and PUT2939165448, respectively, were used to represent these wheat branches. The evolutionary distances are presented in number of base substitutions per site. In constructing the tree, all gaps and missing data were eliminated from the sequence alignment. Genes that do not follow expected topology are found in grey. Labelling of each node was based on Figure 2. Barley genes are highlighted with a box


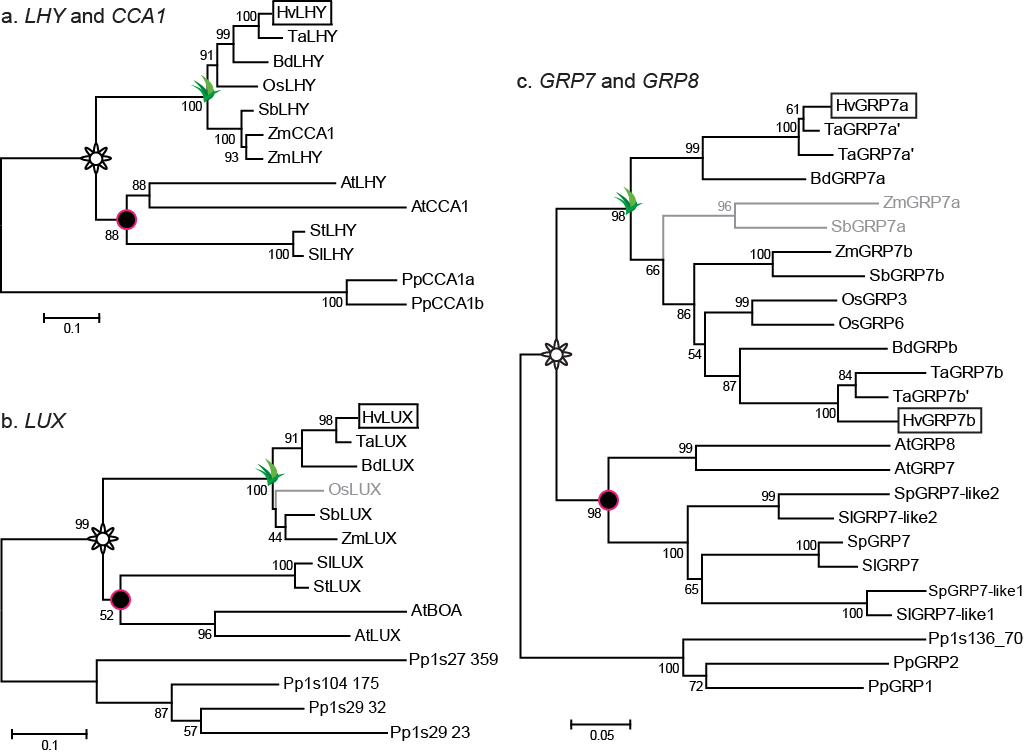


**Fig. S2** Phylogenetic trees of *LHY*, *CCA1*, *LUX*, *GRP7* and *GRP8* genes. a) *LHY* and *CCA1* tree. b) Phylogenetic tree of *LUX* genes identified by cross-species reciprocal BLAST searches. Due to the lack of complete CDS data for the *TaLUX* gene, the partial related CDS from PUT0106334 was used to represent wheat *LUX*. In constructing trees a) and b), all gaps and missing data were eliminated from sequence alignments. Evolutionary distances are presented in number of base substitutions per site. c) *GRP* genes. In constructing the tree, all gaps and missing data were deleted from each pairwise sequence alignment. Evolutionary distances are presented in number of base differences per site. Genes that do not follow expected topology are shown in grey. Labelling of each node was based on Figure 2. Barley genes are highlighted with a box

**
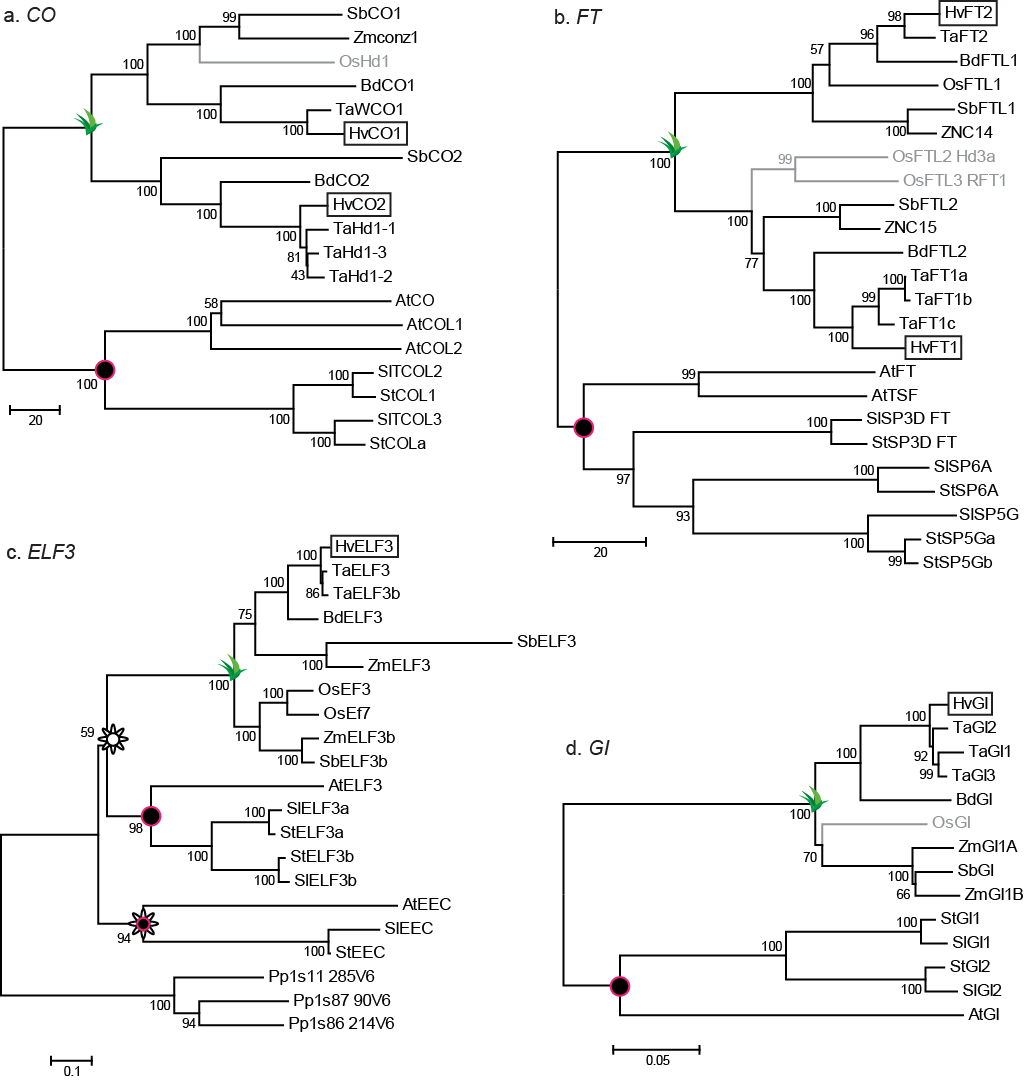
**

**Fig. S3** Phylogenetic trees of *CO*, *FT*, *ELF3* and *GI* genes. a) Subfamily of *COL* genes from Group I, which includes *AtCO*. b) Genes of the *FT* subgroup. c) *ELF3* and *EEC* genes. In constructing the tree, all gaps and missing data were eliminated from the *CO*, *FT* and *ELF3* sequence alignments. d) *GI* genes. In constructing the tree, all gaps and missing data were eliminated from each pairwise sequence alignment. *P. patens* does not contain a true orthologue of *FT*, *CO*, or *GI* and therefore the root was placed on the dicot *FT*, *CO*, and *GI* branches, respectively. The evolutionary distances are presented in number of base differences per site. Genes that do not follow expected topology are found in grey. Labelling of each node was based on Figure 2. Barley genes are highlighted with a box

**Table S1.** The database resources of 10 plant genome sequences analysed in this work.

| Species | Common name | Label on  genes | Genomic database | Annotation Version ^a^ |
| --- | --- | --- | --- | --- |
| *Arabidopsis thaliana* | thale cress | At | The Arabidopsis Information Resource (www.arabidopsis.org) ([Lamesch et al. 2011](#_ENREF_34)) | TAIR10 |
| *Brachypodium distachyon* | purple false brome | Bd | The Munich Information Center for Protein Sequences (http://mips.helmholtz-muenchen.de/plant/) ([Initiative 2010](#_ENREF_28)) | MIPS/JGI Bradi release 1.2 |
| *Hordeum vulgare* cultivar Morex | barley | Hv | Barley Morex assembly3 ([Mayer et al. 2012](#_ENREF_35)).  (http://penguin/ngs/cereals_blast_page.html) | N/A |
| *Oryza sativa* ssp. *japonica* | rice | Os | MSU Rice Genome Annotation Project Database and Resource (http://rice.plantbiology.msu.edu) ([Ouyang et al. 2007](#_ENREF_45)) | MSU RGAP Release 7.0 |
| *Physcomitrella patens* ssp. *patens* | moss | Pp | Phytozome (http://www.phytozome.net/) ([Rensing et al. 2008](#_ENREF_48)) | JGI v1.6 |
| *Solanum lycopersicum* | tomato | Sl | The International Tomato Genome Sequencing Consortium (http://solgenomics.net). ([Consortium 2012](#_ENREF_11)) | ITAG Release 2.3 |
| *Solanum tuberosum* Group *Phureja DM1-3 516 R44* | potato | St | Potato Genome Sequencing Consortium (http://www.potatogenome.net) ([Consortium et al. 2011](#_ENREF_10)) | PGSC_DM_v3.4_gene or PGSC_DM_v3_scaffolds ^*^ |
| *Sorghum bicolor* | sorghum | Sb | The Munich Information Center for Protein Sequences (http://mips.helmholtz-muenchen.de/plant/) ([Paterson et al. 2009](#_ENREF_46)) | MIPS/JGI Sbi release 1.4 |
| *Triticum aestivum* | wheat | Ta | TaGDB (http://www.plantgdb.org/TaGDB/) | GenBank v175 or  **P**lantGDB-assembled **u**nique  **t**ranscripts (PUTs) v163b |
| *Zea mays* ssp. *mays* | maize | Zm | The Phytozome (http://www.phytozome.net/). Line Mo17. Unpublished data produced by the Maize Genome Sequencing Project. | Zmb73 v2 release 5b.60, |

^a^ When necessary, (re)annotation of genomic sequences was performed (detailed in Materials and Methods).

* A few genes of potato have not been annotated but their sequence is present in the DM scaffold data version 3.

N/A: Not available.

**Table S2.** Homologues of *LHY* and *CCA1*, *LUX*, *ELF3*, and *GI* in different land plant species. Where genes have been described previously in the various species, references are given (except in Arabidopsis).

|  | *LHY* and *CCA1* | *LUX* | *ELF3* | *GI* |
| --- | --- | --- | --- | --- |
| *Arabidopsis thaliana* | At1g01060 (*LHY*)  At2g46830 (*CCA1*) | At3g46640 (LUX)  At5g59570 (BOA) | At2g25930 | At1g22770 |
| *Zea mays* | GRMZM2G014902 (*ZmCCA1*) ([Wang et al. 2011](#_ENREF_55))  GRMZM2G474769 (*ZmLHY*) ^a^  ([Hayes et al. 2010](#_ENREF_23)) | GRMZM2G067702  ([Khan et al. 2010](#_ENREF_31)) | GRMZM2G045275 (*ZmELF3b*)  AC233870 (*ZmELF3*) | GRMZM2G107101 (*GI1A*)  GRMZM5G844173 (*GI1B*)  ([Hayes et al. 2010](#_ENREF_23)) |
| *Brachypodium*  *distachyon* | Bradi3g16515  ([Higgins et al. 2010](#_ENREF_25)) | Bradi2g62067  ([Campoli et al. 2013](#_ENREF_6)) | Bradi2g14290  ([Higgins et al. 2010](#_ENREF_25)) | Bradi2g05226  (*BdGI*) ([Hong et al. 2010](#_ENREF_27)) |
| *Sorghum bicolor* | Sb7g003870  ([Murphy et al. 2011](#_ENREF_38)) | Sb03g047330  ([Campoli et al. 2013](#_ENREF_6)) | Sb09g030700 (*ELF3*)  ([Zakhrabekova et al. 2012](#_ENREF_58))  Sb03g025560 (*ELF3b*)  ([Higgins et al. 2010](#_ENREF_25)) | Sb03g003650 (*GI*)  ([Bhosale et al. 2012](#_ENREF_4)) |
| *Oryza sativa* | LOC_Os8g06110  ([Murakami et al. 2007](#_ENREF_37)) | LOC_Os01g74020  ([Murakami et al. 2007](#_ENREF_37)) | LOC_Os01g38530 (*OsEF3*)  ([Fu et al. 2009](#_ENREF_18))  LOC_Os06g05060 (*EF7*)  ([Murakami et al. 2007](#_ENREF_37); [Saito et al. 2012](#_ENREF_49)) | LOC_Os01g08700  ([Hayama et al. 2002](#_ENREF_22)) |
| *Hordeum vulgare* | Hvcontig_51288/1567295 (*HvCCA1* on([Faure et al. 2012](#_ENREF_17)) ^a^ | Hvcontig_2548416  (*HvLUX1* on([Campoli et al. 2013](#_ENREF_6)) | Hvcontig_80895/67536 ([Faure et al. 2012](#_ENREF_17); [Zakhrabekova et al. 2012](#_ENREF_58)) | Hvcontig_58270/1580005  ([Dunford et al. 2005](#_ENREF_15)) |
| *Triticum aestivum* | TAcdna_241985162  ([Campoli et al. 2012b](#_ENREF_7)) | Ta_PUT0106334 ^c^ | TAcdna_118767202 (*TaELF3*) *  ([Faure et al. 2012](#_ENREF_17))  TAcdna_241985055 (*TaELF3b*) * | TAcdna_33333146 (*GI1)*  ([Zhao et al. 2005](#_ENREF_60)) *  TAcdna_50593493 (*GI2*) *  TAcdna_50593495 (*GI3*) * |
| *Solanum tuberosum* | PGSC0003DMG400011294 ^a^ | PGSC0003DMG400002144  ([Campoli et al. 2013](#_ENREF_6)) | PGSC0003DMG400013826  (*ELF3a*)  PGSC0003DMG400029303  (*ELF3b*) | PGSC0003DMS000000006  (*GI1*) ^b^  PGSC0003DMG400018791  (*GI2*) ^a^ |
| *S. lycopersicum* | Solyc10g005080 | Solyc06g005680  ([Campoli et al. 2013](#_ENREF_6)) | Solyc08g065870 (*ELF3a*)  Solyc12g095900 (*ELF3b*) | Soly04g071990 (*GI1*)  Soly12g056650 (*GI2*) |
| *P. patens*  ([Holm et al. 2010](#_ENREF_26)) | Pp1s325_68 (*PpCCA1a*)  Pp1s96_165 (*PpCCA1b*)  ([Okada et al. 2009a](#_ENREF_43); [Okada et al. 2009b](#_ENREF_44)) | Pp1s27_359  Pp1s104_175  Pp1s29_23  Pp1s29_32 | Pp1s86_214  Pp1s11_285  Pp1s87_90 | **None** |

* Alleles, sequenced in hexaploid wheat, are a putative homoeoallelic series on the same group of chromosomes.

^a^ re-annotated; ^b^ annotated; ^c^ partial sequence.

Table S3. Homologues of the pseudo-response regulator genes *TOC1*, *PRR5(9)* and *PRR9(5)*, *PRR7(3)* and *PRR3(7)* in different land plant species. Where genes have been described previously in the various species, references are given (except in Arabidopsis).

|  | *TOC1* | *PRR5(9)* and *PRR9(5)* | *PRR3(7)* and *PRR7(3)* |
| --- | --- | --- | --- |
| *Arabidopsis thaliana* | At5g61380 | At5g24470 (*PRR5*)  At2g46790 (*PRR9*) | At5g60100 (*PRR3*)  At5g02810 (*PRR7*) |
| *Zea mays* | GRMZM2G020081 (*TOC1*)  ([Wang et al. 2011](#_ENREF_55))  GRMZM2G148453 ^a^  (*TOC1b*) ([Hayes et al. 2010](#_ENREF_23)) | GRMZM2G135446 (*PRR59*)  ([Hayes et al. 2010](#_ENREF_23))  GRMZM2G179024 (*PRR95*)  ([Campoli et al. 2012b](#_ENREF_7)) | GRMZM2G095727 (*PRR73*) ([Hayes et al. 2010](#_ENREF_23))  GRMZM2G033962 (*PRR37*) ([Hayes et al. 2010](#_ENREF_23)) ^a^  GRMZM2G005732 (PG) ^a^ |
| *Brachypodium distachyon* ([Higgins et al. 2010](#_ENREF_25)) | Bradi3g48880 | Bradi4g24967 (*PRR59*)  Bradi4g36077 (*PRR95*) | Bradi1g65910 (*PRR73*)  Bradi1g16490 (*PRR37*) |
| *Sorghum bicolor*  ([Takata et al. 2010](#_ENREF_52)) | Sb4g026190  ([Murphy et al. 2011](#_ENREF_38)) | Sb5g003660 (*PRR59*)  Sb2g030870 (*PRR95*) ^a^ | Sb1g038820 (*PRR73*)  Sb6g014570 (*PRR37*) ([Murphy et al. 2011](#_ENREF_38)) |
| *Oryza sativa*  ([Murakami et al. 2007](#_ENREF_37)) | LOC_Os2g40510 | LOC_Os11g05930 (*PRR59*)  LOC_Os9g36220 (*PRR95*) | LOC_Os3g17570 (*PRR73*)  LOC_Os7g49460 (*PRR37*) |
| *Hordeum vulgare*  ([Campoli et al. 2012b](#_ENREF_7)) | Hvcontig_37494  ([Faure et al. 2012](#_ENREF_17)) | Hvcontig_46739 (*PRR59*)  Hvcontig_41351 (*PRR95*)  ([Higgins et al. 2010](#_ENREF_25)) | Hvcontig_1563982 (*PRR73*) ([Higgins et al. 2010](#_ENREF_25))  Hvcontig_94710 (*PPD-H1*/*PRR37*)  ([Jones et al. 2008](#_ENREF_29); [Turner et al. 2005](#_ENREF_53)) |
| *Triticum aestivum* | TAcdna_241985932  ([Campoli et al. 2012b](#_ENREF_7)) | Ta_PUT2939165448 (*PRR59*) ^c^  TAcdna_241983556 (*PRR95*)  ([Campoli et al. 2012b](#_ENREF_7)) | Ta_PUT18538 (*PRR73*) ^c^  *PPDD1*, *PPDB1*, *PPDA1*  (*PRR37*s) ([Beales et al. 2007](#_ENREF_1)) * |
| *Solanum tuberosum* | PGSC0003DMG400033048 (*TOC1*) ^a^ PGSC0003DMG400019518 (*TOC1-like*) | PGSC0003DMG400000584 (*PRR5*)  PGSC0003DMG402011297 (*PRR9*) | PGSC0003DMS000000129 (*PRR3*) ^b^  PGSC0003DMS000000068 (*PRR7*) ^a^ |
| *S. lycopersicum* | Solyc06g069690 (*TOC1*)  Solyc03g115770 (*TOC1-like*) | Solyc03g081240 (*PRR5*)  Solyc10g005030 (*PRR9*) | Solyc04g049670/80 (*PRR3*) ^a^  Solyc10g086000 (*PRR7*) |
| *P. patens* | Pp1s412_23 (*PpPRR1*), Pp1s81_131 (*PpPRR2*), Pp1s412_35 (*PpPRR3*), Pp1s81_144 (*PpPRR4*) ([Holm et al. 2010](#_ENREF_26); [Satbhai et al. 2010](#_ENREF_50)) | | |

* Three alleles, sequenced in hexaploid wheat, are a homoeoallelic series on the group 2 chromosomes. Sequences retrieved from

literature ([Beales et al. 2007](#_ENREF_1)).

^a^ re-annotated; ^b^ annotated; ^c^ partial sequence; ^PG^ pseudo-gene.

Table S4. Homologues of *ZTL*, *FKF1*, *GRP7* and *GRP8* genes in different land plant species. Where genes have been described previously in the various species, references are given (except in Arabidopsis).

|  | *ZTL* | *FKF1* | *GRP7* and *GRP8* |
| --- | --- | --- | --- |
| *Arabidopsis thaliana* | At5g57360 (*ZTL*)  At2g18915 (*LKP2*) | At1g68050 | At2g21660 (*GRP7*)  At4g39260 (*GRP8*) |
| *Zea mays* | GRMZM2G113244 (*ZmZTLa*)  GRMZM2G147800 (*ZmZTLb*)  GRMZM2G115914 (PG) | GRMZM2G106363 (*ZmFKF1a*)  GRMZM2G107945 (*ZmFKF1b*)  ([Hayes et al. 2010](#_ENREF_23)) | GRMZM2G165901 (*GRP7a*)  GRMZM2G080603 (*GRP7b*) |
| *Brachypodium*  *distachyon* | Bradi1g33610 (*BdZTLa*)  Bradi3g04040 (*BdZTLb*)  ([Higgins et al. 2010](#_ENREF_25)) | Bradi4g16630  ([Higgins et al. 2010](#_ENREF_25)) | Bradi1g12787 (*GPR7a*)  Bradi4g00940 (*GRP7b*) |
| *Sorghum bicolor* | Sb10g028340 (*SbZTLa*)  Sb04g003660 (*SbZTLb*) | Sb05g021030 | Sb08g022740 (*GRP7a*)  Sb01g012300 (*GRP7c*) |
| *Oryza sativa* | LOC_Os02g05700 (*OsFBO08*/*ZTL1*) LOC_Os06g47890 (*OsFBO09*/*ZTL2*)  ([Murakami et al. 2007](#_ENREF_37)) | LOC_Os11g34460  ([Higgins et al. 2010](#_ENREF_25); [Murakami et al. 2007](#_ENREF_37)) | LOC_Os03g46770 (*GRP3*)  LOC_Os12g43600 (*GRP6*)  ([Kim et al. 2010](#_ENREF_32)) |
| *Hordeum vulgare* | Hvcontig_273830 (*HvZTLa*)  Hvcontig_158755 (*HvZTLb*) | Hvcontig_38586 | Hvcontig_1578172 (*GRP7a*) ^a^  ([Campoli et al. 2012b](#_ENREF_7))  Hvcontig_43832/46175 (*GRP7b*) ^a^ |
| *Triticum aestivum* | TAcdna_241984947 (*TaZTLa*)  Ta_PUT43520 (*TaZTLb*) ^c^ | TAcdna_118767204 | Tacdna_241988564 (*GRP7a*) and  Tacdna_114145393 (*GRP7a’*)  Tacdna_974604 (*GRP7b*) and  Tacdna_241988180 (*GRP7b’*) |
| *Solanum tuberosum* | PGSC0003DMS000000971 ^b^ | PGSC0003DMG400019971 | PGSC0003DMG400000708 (*GRP7*)  PGSC0003DMG400033902 (*GRP7-like1*)  PGSC0003DMG400033903 (*GRP7-like2*) |
| *S. lycopersicum* | Solyc07g017750 ^a^ | Solyc01g005300 ^a^ | Solyc01g109660 (*GRP7*)  Solyc10g051380 (*GRP7-like1*)  Solyc10g051390 (*GRP7-like2*) |
| *P. patens* | **None** ([Holm et al. 2010](#_ENREF_26)) | **None** ([Holm et al. 2010](#_ENREF_26)) | Pp1s42_251 (*GRP1*), Pp1s123_58 (*GRP2)*  ([Nomata et al. 2004](#_ENREF_42))  Pp1s136_70 |

^a^ re-annotated; ^b^ annotated; ^c^ partial sequence; ^PG^ pseudo-genes.

Table S5. Dicot-specific homologues of *ELF4* and *EEC* in three dicotyledonous plant species and the Arabidopsis-specific *CHE* and *TSF*.

|  | *ELF4* | *EEC* | *CHE* | *TSF* |
| --- | --- | --- | --- | --- |
| *Arabidopsis thaliana* | At2g40080 (*ELF4*)  At2g29950 (*ELF4-like1*) | At3g21320 | At5g08330 | At4g20370 |
| *Solanum tuberosum* | PGSC0003DMG400006624 (*ELF4*)  PGSC0003DMG400001221 (*ELF4-like6*)  PGSC0003DMG400030357 (*ELF4-like5*) | PGSC0003DMG400004837 | **None** | **None** |
| *S. lycopersicum* | Solyc06g051660 (*ELF4*)  Solyc11g028200 (*ELF4-like5a*)  Solyc06g076960 (*ELF4-like5b*)  scf7180001945491 ^PG^ | Solyc06g062480 | **None** | **None** |

^PG^ Tomato pseudogene: sequence annotated in the Tomato WGS Alternate Scaffolds cabog1.00.

Table S6. Homologues of *FT*, *ELF4-like* and *CO* genes in different land plant species. Where genes have been described previously in the various species, references are given (except in Arabidopsis).

|  | *CO* | *FT* | *ELF4-like2/3/4* |
| --- | --- | --- | --- |
| *Arabidopsis*  *thaliana* | At5g15840 (*CO*), At5g15850 (*COL1*)  At3g02380 (*COL2*) | At1g65480 | At2g06255 (*ELF4-like3*), At1g17455 (*ELF4-like4*), At1g72630 (*ELF4-like2*) |
| *Zea mays* | GRMZM2G405368 (*CONZ1*)  ([Miller et al. 2008](#_ENREF_36))  *ZmCO2* ^PG^ | GRMZM2G373928 (*ZNC14*)  GRMZM2G051338 (*ZCN15*)  ([Danilevskaya et al. 2008](#_ENREF_13)) | GRMZM5G877647 (*ELF4-like3*)  GRMZM2G382774 (*ELF4-likeB1*)  GRMZM2G359322 (*ELF4-likeB2*)  GRMZM2G025646 (*ELF4*) ([Zhang 2011](#_ENREF_59)) |
| *Brachypodium*  *distachyon* | Bradi1g43670 (*CO1*), Bradi3g56260 (*CO2*)  ([Higgins et al. 2010](#_ENREF_25)) | Bradi2g07070 (*FTL1*), Bradi1g48830 (*FTL2*)  ([Higgins et al. 2010](#_ENREF_25)) | Bradi4g13227 (*ELF4-like3*), Bradi4g29580 (*ELF4-likeA*), Bradi1g60090 (*ELF4-likeB*) |
| *Sorghum*  *bicolor* | Sb10g010050 (*CO1*)  ([Murphy et al. 2011](#_ENREF_38))  Sb04g029180 (*CO2*) ^c^ | Sb10g003940 (*FTL2*) ([Murphy et al. 2011](#_ENREF_38))  Sb03g001700 (*FTL1*) | Sb05g025110 (*ELF4-like3*)  Sb02g023990 (*ELF4-likeA*)  Sb01g032750 (*ELF4-likeB*) |
| *Oryza sativa* | LOC_Os06g16370 (*HD1* or *OsA*)  ([Cockram et al. 2012](#_ENREF_9); [Yano et al. 2000](#_ENREF_57)) | LOC_Os06g06300 (*FTL3_RFT1*)  LOC_Os06g06320 (*FTL2_ Hd3a*)  LOC_Os01g11940 (*FTL1*)  ([Faure et al. 2007](#_ENREF_16)) | LOC_Os11g40610 (*ELF4-like3*)  LOC_Os03g29680 (*ELF4-likeB*)  LOC_Os08g27860 (*ELF4-likeA1*)  LOC_Os08g27870 (*ELF4-likeA2*) |
| *Hordeum vulgare* | Hvcontig_138334 (*CO1*)  ([Campoli et al. 2012a](#_ENREF_5))  Hvcontig_6805 (*CO2*)  ([Griffiths et al. 2003](#_ENREF_21)) | Hvcontig_54983 (*FT1* or *VRN-H3*)  ([Yan et al. 2006](#_ENREF_56))  Hvcontig_1558556/136243 (*FT2*)  ([Faure et al. 2007](#_ENREF_16)) | Hvcontig_42805 (*ELF4-likeA*)  ([Kolmos et al. 2009](#_ENREF_33))  Hvcontig_58806 (*ELF4-like3*) |
| *Triticum*  *aestivum* | Tacdna_169807975 (*WCO1*) ([Shimada et al. 2009](#_ENREF_51))  Tacdna_36789816 (*HD1-3*) *,  GenBank_AB094488 (*HD1-2*)* ^c^ and  Tacdna_36789805 (*HD1-1*)* ([Nemoto et al. 2003](#_ENREF_41)) | Tacdna_169807973 (*FT1a*)*,  Tacdna_40644759 (*FT1b*) ^a^ *  and Tacdna_56694631 (*FT1c*) *  Tacdna_ 32128602 (*FT2*) ([Yan et al. 2006](#_ENREF_56)) | TaPUT_145474 (*ELF4-like3*) ^a^  TaPUT_3048165449 (*ELF4-likeA*) |
| *Solanum*  *tuberosum* | PGSC0003DMG402010056 (*COL1*)  PGSC0003DMG401010056 (*COLa*)  ([González-Schain et al. 2012](#_ENREF_19)) | PGSC0003DMG400023365 (*SP6A*)  ([Consortium et al. 2011](#_ENREF_10); [Initiative 2010](#_ENREF_28))  PGSC0003DMB000000142 (*FT_SP3D*)  ([Navarro et al. 2011](#_ENREF_39)) PGSC0003DMB00512  (*SP5Ga* and *SP5Gb*) ^b^ | PGSC0003DMG400002144 (*ELF4-like3*)  PGSC0003DMG400009846 (*ELF4-like8*)  PGSC0003DMG400011596 (*ELF4-like7*) |
| *S. lycopersicum* | Solyc02g089540 (*TCOL2*)  Solyc02g089520 (*TCOL3*)  ([Ben-Naim et al. 2006](#_ENREF_3)) | Solyc05g055660 (*SP6A*), Solyc03g063100  (*FT_SP3D*) Solyc05g053850 (*SP5G*)  ([Carmel-Goren et al. 2003](#_ENREF_8)) | Solyc07g041340 (*ELF4-like3*)  Solyc12g049290 (*ELF4-like7*) |
| *P. patens* | None | **None** ([Hedman et al. 2009](#_ENREF_24); [Karlgren et al. 2011](#_ENREF_30)) | Pp1s180_31 (*ELF4-like3*) |

* Three alleles, sequenced in hexaploid wheat, are derived from three homoeologous genomes.

^a^ partial sequence; ^b^ annotated; ^c^ re-annotated; ^PG^ pseudogene.

**Note 1. Likely incorrect gene duplication events**

Gene duplications have been previously reported in the literature but were not confirmed in our analysis.

**Barley ZTL**

In barley, a *ZTL*-type gene called *HvDRF* was identified and found to be involved in disease resistance ([Dagdas et al. 2009](#_ENREF_12)). The sequence available for this gene on GenBank (FJ913271) codes for a protein highly similar to *AtZTL* and *TaZTLa*. However, phylogenetic analysis demonstrates that *HvDRF* forms a sister branch with all monocots rather than being positioned close to *HvZTLa* and *TaZTLa* (data not shown), which is incongruent with phylogeny. Moreover, cross-species reciprocal BLASTs using this sequence did not retrieve any orthologue in any species. The genomic sequence for this gene is not available in any database, and therefore, the evolutionary history of *HvDRF* and its relation with *HvZTLa* and *HvZTLb* cannot be determined at this time.

**ELF3 in Pooideae**

*ELF3* appears to be present as a single copy in Pooideae species but Yang *et al*. (2013) suggested that *Brachypodium*, which belongs to Pooideae, has two *ELF3* homologues. However, only one gene (*BdELF3* - Bradi2g14290) was presented in their phylogenetic tree and we identified the same gene as a single gene in our cross-species reciprocal BLASTs.

**LUX in monocots**

One study suggested that there are two *LUX* genes in monocots, *LUX1* and *LUX2* ([Campoli et al. 2013](#_ENREF_6)). The authors propose that gene duplications occurred independently in the evolution of monocots (*LUX1* and *LUX2*) and Arabidopsis (*LUX* and *BOA*). The sequence available for *HvLUX1* on GenBank (BAJ88719) was identical to *HvLUX* identified by cross-species reciprocal BLAST analysis using *AtLUX* (At3g46640). On the other hand, reciprocal BLASTX using *HvLUX2* cDNA against the Arabidopsis protein database identified different genes. The top three hits were the transcription factors At3g10760, At2g40970 and At5g050090, which all identify *HvLUX2* in GenBank. Moreover, phylogenetic analysis of the *LUX* superfamily on the Plaza database showed separate clusters of *LUX* genes from land plants, indicating that their common ancestor had both *LUX1* and *LUX2* genes. In Arabidopsis, *LUX2* was duplicated twice and is now represented as the three transcription factors mentioned above (At3g10760, At2g40970 and At5g050090). Therefore, it is likely that *HvLUX2* belongs to a different subfamily of *LUX*-related genes, which have evolutionarily diverged from *HvLUX1* since speciation in land plants and would explain the extensive distance between *LUX1* and *LUX2* clades observed by Campoli *et al.* (2013), as well as the inconsistencies in phylogeny.

**Note 2. Arabidopsis-specific clock-associated genes *AtCHE* and *AtCAB2***

***AtCHE***

Cross-species reciprocal BLAST using the single exon gene *AtCHE* did not identify orthologues in any of the species analysed. However, another Arabidopsis gene, At5g23280 (*AtTCP7*), was identified by reciprocal BLAST and identified orthologues in all species analysed, as well as several paralogues (data not shown). *AtCHE* is one of its paralogues, present only in Arabidopsis (of all species analysed here). In fact, *AtCHE* represents a recent duplication (~50 Mya, from the α duplication event) of *AtTCP7* (At5g23280) in Arabidopsis ([Navaud et al. 2007](#_ENREF_40)) and thus, is not expected to be present in the other plant species analysed in this study. Higgins *et al.* (2010) describes a *CHE* gene in *Brachypodium*, Bradi3g60350 (*BdCHE*), but cross-species reciprocal BLAST suggests that this is not the case. *AtTCP7* belongs to an angiosperm-specific subfamily of *TCP*s ([Navaud et al. 2007](#_ENREF_40)) and is likely to be the real orthologue of *BdCHE*.

***AtCAB2***

Similarly, orthologues of *AtCAB2* could not be identified in any species analysed. This gene belongs to a large family of light-harvesting CHLOROPHYLL A/B-BINDING proteins (CAB) whose evolution has been highly debated but it is suggested that each taxa, i.e. moss, monocots, and dicots, might have evolved their own *CAB* genes independently during their evolution ([Dittami et al. 2010](#_ENREF_14); [Green 2001](#_ENREF_20); [Umate 2010](#_ENREF_54)). Arabidopsis has 5 genes that belong to this family, while *Brachypodium* has 4 genes, maize has 6 genes, sorghum has 5 genes and rice has 3 genes ([Plaza 2011](#_ENREF_47)). Barley and wheat have at least 17 gene members (data not shown). In particular, *HvCABa* is represented in the array feature baak26h09, which was identified as being differentially expressed in the barley clock mutant *eam8* (*elf3* loss-of-function), when compared with WT (Faure et al 2012). This gene is a hitherto uncharacterised paralogue of the barley *CAB* family. Other *HvCAB* members have been identified and analysed elsewhere ([Beator et al. 1992](#_ENREF_2); [Campoli et al. 2012b](#_ENREF_7); [Faure et al. 2012](#_ENREF_17)). The complex results from cross-species reciprocal BLAST analysis did not allow the identification of a true *CAB2* orthologue.

**Supplementary References**

Beales J, Turner A, Griffiths S, Snape JW, Laurie DA (2007) A *pseudo-response regulator* is misexpressed in the photoperiod insensitive *Ppd-D1a* mutant of wheat (*Triticum aestivum* L.). Theor Appl Genet 115:721-733

Beator J, Pötter E, Kloppstech K (1992) The effect of heat shock on morphogenesis in barley : coordinated circadian regulation of mRNA levels for light-regulated genes and of the capacity for accumulation of chlorophyll protein complexes. Plant Physiol 100:1780-1786

Ben-Naim O et al. (2006) The CCAAT binding factor can mediate interactions between CONSTANS-like proteins and DNA. Plant J 46:462-476

Bhosale SU et al. (2012) Association analysis of photoperiodic flowering time genes in West and Central African sorghum [*Sorghum bicolor* (L.) Moench]. BMC Plant Biol 12:32

Campoli C, Drosse B, Searle I, Coupland G, von Korff M (2012a) Functional characterisation of *HvCO1*, the barley (*Hordeum vulgare*) flowering time ortholog of *CONSTANS*. Plant J 69:868-880

Campoli C, Pankin A, Drosse B, Casao CM, Davis SJ, von Korff M (2013) *HvLUX1* is a candidate gene underlying the *early maturity 10* locus in barley: phylogeny, diversity, and interactions with the circadian clock and photoperiodic pathways. New Phytol 199:1045-1059

Campoli C, Shtaya M, Davis SJ, von Korff M (2012b) Expression conservation within the circadian clock of a monocot: natural variation at barley *Ppd-H1* affects circadian expression of flowering time genes, but not clock orthologs. BMC Plant Biol 12:97

Carmel-Goren L, Liu YS, Lifschitz E, Zamir D (2003) The *SELF-PRUNING* gene family in tomato. Plant Mol Biol 52:1215-1222

Cockram J, Thiel T, Steuernagel B, Stein N, Taudien S, Bailey PC, O'Sullivan DM (2012) Genome dynamics explain the evolution of flowering time CCT domain gene families in the Poaceae. PLoS One 7:e45307

Consortium PGS et al. (2011) Genome sequence and analysis of the tuber crop potato. Nature 10:189-195

Consortium TTG (2012) The tomato genome sequence provides insights into fleshy fruit evolution. Nature 485

Dagdas YF, Dagdas G, Unver T, Akkaya MS (2009) A new ZTL-type F-box functions as a positive regulator in disease resistance: VIGS analysis in barley against powdery mildew. Physiol Mol Plant Pathol 74:41-44

Danilevskaya ON, Meng X, Hou Z, Ananiev EV, Simmons CR (2008) A genomic and expression compendium of the expanded PEBP gene family from maize. Plant Physiol 146:250-264

Dittami SM, Michel G, Collén J, Boyen C, Tonon T (2010) Chlorophyll-binding proteins revisited – a multigenic family of light-harvesting and stress proteins from a brown algal perspective. BMC Evol Biol 10:365

Dunford RP, Griffiths S, Christodoulou V, Laurie DA (2005) Characterisation of a barley (*Hordeum vulgare* L.) homologue of the *Arabidopsis* flowering time regulator GIGANTEA. Theor Appl Genet 110:925-931

Faure S, Higgins J, Turner A, Laurie DA (2007) The FLOWERING LOCUS T-like gene family in barley (Hordeum vulgare). Genetics 176:599-609

Faure S, Turner AS, Gruszka D, Christodoulou V, Davis SJ, von Korff M, Laurie DA (2012) Mutation at the circadian clock gene *EARLY MATURITY 8* adapts domesticated barley (*Hordeum vulgare*) to short growing seasons. Proc Natl Acad Sci USA 109:8328-8333

Fu C, Yang XO, Chen X, Chen W, Ma Y, Hu J, Li S (2009) *OsEF3*, a homologous gene of *Arabidopsis* ELF3, has pleiotropic effects in rice. Plant Biol 11:751-757

González-Schain ND, Díaz-Mendoza M, Zurczak M, Suárez-López (2012) Potato CONSTANS is involved in photoperiodic tuberization in a graft-transmissible manner. Plant J 70:678-690

Green BR (2001) Was "molecular opportunism" a factor in the evolution of different photosynthetic light-harvesting pigment systems? Proc Natl Acad Sci USA 98:2119-2121

Griffiths S, Dunford RP, Coupland G, Laurie DA (2003) The evolution of CONSTANS-like gene families in barley, rice, and Arabidopsis. Plant Physiol 131:1855-1867

Hayama R, Izawa T, Shimamoto K (2002) Isolation of rice genes possibly involved in the photoperiodic control of flowering by a fluorescent differential display method. Plant and Cell Physiology 43:494-504

Hayes KR, Beatty B, Meng X, Simmons CR, Habben JE, Danilevskaya ON (2010) Maize global transcriptomics reveals pervasive leaf diurnal rhythms but rhythms in developing ears are largely limited to the core oscillator. PLoS One 5:e12887

Hedman H, Källman T, Lagercrantz U (2009) Early evolution of the MFT-like gene family in plants. Plant Mol Biol 70:359-369

Higgins JA, Bailey PC, Laurie DA (2010) Comparative genomics of flowering time pathways using *Brachypodium distachyon* as a model for the temperate grasses. PLoS One 5:e10065

Holm K, Källman T, Gyllenstrand N, Hedman H, Lagercrantz U (2010) Does the core circadian clock in the moss *Physcomitrella patens* (Bryophyta) comprise a single loop? BMC Plant Biol 10:109

Hong SY, Lee S, Seo PJ, Yang MS, Park CM (2010) Identification and molecular characterization of a Brachypodium distachyon GIGANTEA gene: functional conservation in monocot and dicot plants. Plant Mol Biol 72:485-497

Initiative IB (2010) Genome sequencing and analysis of the model grass Brachypodium distachyon. Nature 463:763-768

Jones H et al. (2008) Population-based resequencing reveals that the flowering time adaptation of cultivated barley originated east of the Fertile Crescent. Mol Biol Evol 25:2211-2219

Karlgren A, Gyllenstrand N, Källman T, Sundström JF, Moore D, Lascoux M, Lagercrantz U (2011) Evolution of the PEBP Gene Family in Plants: Functional Diversification in Seed Plant Evolution. Plant Physiol 156:1967-1977

Khan S, Rowe SC, Harmon FG (2010) Coordination of the maize transcriptome by a conserved circadian clock. BMC Plant Biol 10:126

Kim JY, Kim WY, Kwak KJ, Oh SH, Han YS, Kang H (2010) Glycine-rich RNA-binding proteins are functionally conserved in *Arabidopsis thaliana* and *Oryza sativa* during cold adaptation process. J Exp Bot 61:2317-2325

Kolmos E et al. (2009) Integrating ELF4 into the circadian system through combined structural and functional studies. HFSP Journal 3:350-366

Lamesch P et al. (2011) The Arabidopsis Information Resource (TAIR): improved gene annotation and new tools. Nucleic Acids Res 40:D1202-1210

Mayer KF et al. (2012) A physical, genetic and functional sequence assembly of the barley genome. Nature 491:711-716

Miller TA, Muslin EH, Dorweiler JE (2008) A maize CONSTANS-like gene, conz1, exhibits distinct diurnal expression patterns in varied photoperiods. Planta 227:1377-1388

Murakami M, Tago Y, Yamashino T, Mizuno T (2007) Comparative overviews of clock-associated genes of *Arabidopsis thaliana* and *Oryza sativa*. Plant and Cell Physiology 48:110-121

Murphy RL et al. (2011) Coincident light and clock regulation of *pseudoresponse regulator protein 37* (*PRR37*) controls photoperiodic flowering in sorghum. Proc Natl Acad Sci USA 108:16469-16474

Navarro C et al. (2011) Control of flowering and storage organ formation in potato by FLOWERING LOCUS T. Nature 478:119-122

Navaud O, Dabos P, Carnus E, Tremousaygue D, Hervé C (2007) TCP Transcription Factors Predate the Emergence of Land Plants. J Mol Evol 65:23-33

Nemoto Y, Kisaka M, Fuse T, Yano M, Ogihara Y (2003) Characterization and functional analysis of three wheat genes with homology to the CONSTANS flowering time gene in transgenic rice. Plant J 36:82-93

Nomata T, Kabeya Y, Sato N (2004) Cloning and characterization of glycine-rich RNA-binding protein cDNAs in the moss *Physcomitrella patens*. Plant and Cell Physiology 45:48-56

Okada R, Kondo S, Satbhai SB, Yamaguchi N, Tsukuda M, Aoki S (2009a) Functional characterization of CCA1/LHY homolog genes, PpCCA1a and PpCCA1b, in the moss *Physcomitrella patens*. Plant J 60:551-563

Okada R, Satbhai SB, Aoki S (2009b) Photoperiod-dependent regulation of cell growth by PpCCA1a and PpCCA1b genes encoding single-myb clock proteins in the moss *Physcomitrella patens*. Genes Genet Syst 84:379-384

Ouyang S et al. (2007) The TIGR Rice Genome Annotation Resource: improvements and new features. Nucleic Acids Res 35:D883-887

Paterson AH et al. (2009) The *Sorghum bicolor* genome and the diversification of grasses. Nature 457:551-556

A resource for plant comparative genomics (2011).

Rensing SA et al. (2008) The Physcomitrella genome reveals evolutionary insights into the conquest of land by plants. Science 319:64-69

Saito H et al. (2012) Ef7 Encodes an ELF3-like Protein and Promotes Rice Flowering by Negatively Regulating the Floral Repressor Gene Ghd7 under Both Short- and Long-Day Conditions. Plant and Cell Physiology 53:717-728

Satbhai SB et al. (2010) Pseudo-response regulator (PRR) homologues of the moss Physcomitrella patens: insights into the evolution of the PRR family in land plants. DNA Res 18:39-52

Shimada S et al. (2009) A genetic network of flowering-time genes in wheat leaves, in which an APETALA1/FRUITFULL-like gene, VRN1, is upstream of FLOWERING LOCUS T. Plant J 58:668-681

Takata N, Saito S, Saito CT, Uemura M (2010) Phylogenetic footprint of the plant clock system in angiosperms: evolutionary processes of Pseudo-Response Regulators. BMC Evol Biol 10:126

Turner A, Beales J, Faure S, Dunford RP, Laurie D (2005) The Pseudo-Response Regulator Ppd-H1 Provides Adaptation to Photoperiod in Barley. Science 310:1031-1034

Umate P (2010) Genome-wide analysis of the family of light-harvesting chlorophyll a/b-binding proteins in Arabidopsis and rice. Plant Signaling & Behavior 5:1537-1542

Wang X et al. (2011) Robust expression and association of ZmCCA1 with circadian rhythms in maize. Plant Cell Rep 30:1261-1272

Yan L et al. (2006) The wheat and barley vernalization gene VRN3 is an orthologue of FT. Proc Natl Acad Sci USA 103:19581-19586

Yano M et al. (2000) Hd1, a major photoperiod sensitivity quantitative trait locus in rice, is closely related to the Arabidopsis flowering time gene CONSTANS. Plant Cell 12:2473-2484

Zakhrabekova S et al. (2012) Induced mutations in circadian clock regulator *Mat-a* facilitated short-season adaptation and range extension in cultivated barley. Proc Natl Acad Sci USA 109:4326-4331 doi:10.1073/pnas.1113009109

Zhang SF (2011) Cloning and Characterization of Photoperiod Sensitive Gene ZmELF4 in Maize. Master's thesis, Henan Agricultural University

Zhao XY, Liu MS, Li JR, Guan CM, Zhang XS (2005) The wheat TaGI1, involved in photoperiodic flowering, encodes an Arabidopsis GI ortholog. Plant Mol Biol 58:53-64
